# Oxytocin and Related Peptide Hormones: Candidate Anti-Inflammatory Therapy in Early Stages of Sepsis

**DOI:** 10.3389/fimmu.2022.864007

**Published:** 2022-04-29

**Authors:** Syed Faizan Mehdi, Suma Pusapati, Raja Ram Khenhrani, Muhammad Saad Farooqi, Sobia Sarwar, Ahmad Alnasarat, Nimisha Mathur, Christine Noel Metz, Derek LeRoith, Kevin J. Tracey, Huan Yang, Michael J. Brownstein, Jesse Roth

**Affiliations:** ^1^ The Feinstein Institutes for Medical Research/Northwell Health, Manhasset, NY, United States; ^2^ Division of Endocrinology, Diabetes & Bone Disease, Icahn School of Medicine at Mt. Sinai, New York, NY, United States; ^3^ Azevan Pharmaceuticals Inc., Bethlehem, PA, United States

**Keywords:** inflammation, sepsis, oxytocin, anti-inflammatory, cytokine storm

## Abstract

Sepsis is a potentially life-threatening systemic inflammatory syndrome characterized by dysregulated host immunological responses to infection. Uncontrolled immune cell activation and exponential elevation in circulating cytokines can lead to sepsis, septic shock, multiple organ dysfunction syndrome, and death. Sepsis is associated with high re-hospitalization and recovery may be incomplete, with long term sequelae including post-sepsis syndrome. Consequently, sepsis continues to be a leading cause of morbidity and mortality across the world. In our recent review of human chorionic gonadotropin (hCG), we noted that its major properties including promotion of fertility, parturition, and lactation were described over a century ago. By contrast, the anti-inflammatory properties of this hormone have been recognized only more recently. Vasopressin, a hormone best known for its anti-diuretic effect, also has anti-inflammatory actions. Surprisingly, vasopressin’s close cousin, oxytocin, has broader and more potent anti-inflammatory effects than vasopressin and a larger number of pre-clinical studies supporting its potential role in limiting sepsis-associated organ damage. This review explores possible links between oxytocin and related octapeptide hormones and sepsis-related modulation of pro-inflammatory and anti-inflammatory activities.

## Introduction

First recorded in Homer’s ancient Greek poems, the word “sepsis” stems from “sepo”, which means ‘I rot’. This medical term was later used by Hippocrates around 400 BC and Galen before 200 AD. The “Germ Theory” of disease, introduced in the 1800s, suggested that harmful microbes caused sepsis. Subsequently, numerous experimental and clinical trials conducted throughout the twentieth century demonstrated the importance of the host immune response in the manifestations of sepsis. Over the last few decades, additional research and clinical trials have further improved our understanding of the pathophysiology of sepsis and have led to significant improvements in the definition and management of this disorder.

Monetary costs of sepsis– Despite significant advances, sepsis continues to be a major cause of morbidity and mortality in critically ill patients, most recently those with COVID-19 infections. Signs of multi-organ injury typical of sepsis occur in approximately 2-5% of those with COVID-19 after 8-10 days post-infection (1, 2). In a recent update from the Centers for Disease Control and Prevention, 1 in 3 patients who die in the hospital are reported to have had sepsis. In a typical year, almost 1.7 million Americans develop sepsis and approximately 270,000 die as a result of it (3). Furthermore, managing sepsis patients is the most expensive condition treated in hospitals in the United States (4). In 2013, sepsis accounted for more than $24 billion in U.S. hospital expenses, or 13% of total U.S. hospital costs (5).

Many studies have revealed that multiple mammalian peptide hormones including oxytocin, vasopressin, ghrelin, human chorionic gonadotropin (hCG), glucagon, and glucagon-like peptide-1 have anti-sepsis potential (1–6). While vasopressin has significant anti-inflammatory effects, oxytocin exerts broader anti-inflammatory effects and acts on the majority of the organs susceptible to sepsis damage. Moreover, oxytocin appears to be less toxic and causes minimal blood pressure fluctuations when compared to vasopressin. As a result, we have focused on oxytocin for this review. Most notably, oxytocin abolishes the sepsis-induced increase in TNF-α and thereby protects against sepsis-related, cytokine-mediated damage to multiple organs. One proposed mechanism of action is blocking the transition of macrophages from neutral to pro-inflammatory modes (7). Ligation of oxytocin to the oxytocin receptor results in the inhibition of NF-κB signaling (8) and thus decreases the production of TNF-α and other potentially damaging inflammatory cytokines (7, 9). Oxytocin also impedes the endotoxin-induced increases in cortisol, TNF-α, and IL-6 in a rodent model of sepsis (7, 9).

Lipopolysaccharides (LPSs) can promote metabolic endotoxemia, which is considered inflammatory and metabolically detrimental based on Toll-like receptor (TLR)4 agonists. Because of ambiguity we have chosen to avoid LPS as a substitute model for sepsis. We suggest that oxytocin may be very useful for combatting uncontrolled inflammation during sepsis (6–9).

## Emergence of Oxytocin

Henry Dale, a British pharmacologist, first mentioned some actions of pituitary extracts in 1906 (10). Among other things, he reported that pituitary preparations from oxen caused the pregnant uterus of a cat to contract. The factor responsible for this action was subsequently called oxytocin, originating from the Greek word “oxutokia”, meaning ‘sudden delivery’. British physician William Blair-Bell confirmed Dale’s findings in 1909, and showed that posterior pituitary extracts prevented post-partum hemorrhage, an important clinical application of oxytocin (11). After Dale’s and Blair-Bell’s reports, other investigators showed that posterior pituitary extracts increase milk ejection (12, 13). In 1928, Kamm et al. separated several active factors from posterior pituitary extracts, specifically isolating vasopressin and oxytocin from other molecules in the posterior pituitary extracts (14, 15).

In the 1930s using a balloon catheter, it was demonstrated that nipple stimulation induced uterine contractions in women 6–7 days after delivery (16). Subsequently, oxytocin was administered to women with labor dystocia (also known as protracted labor). Because it was administered subcutaneously, oxytocin was considered to be physiological, and hence harmless; the dosages were not standardized and it caused the death of many women and fetuses due to uterine rupture and intrauterine asphyxia, respectively (17). In 1948, Burn et al. introduced a method for slowly delivering a dilute oxytocin solution intravenously and Theobald succeeded in using oxytocin infusions to induce labor safely (18–20).

In 1953, du Vigneaud et al. sequenced and synthesized oxytocin and vasopressin (21–23). Two years later he was awarded a Nobel Prize in chemistry for this work. Scharrer, Scharrer, and Bargmann did pioneering studies of the neuronal sources of the physiologically active posterior pituitary hormones, establishing neuroendocrinology as a new field of study (24). Roth et al. at NIH later demonstrated cell surface receptors specific for oxytocin (25, 26). In 1980, Brownstein et al. showed that oxytocin was produced by the posterior pituitary from a neurophysin-containing precursor (27). Its cDNA was isolated by Ivell and Richter in 1984 (28).

In 1992, the only known receptor for oxytocin was cloned by Brownstein and his colleagues in Okayama’s laboratory (29). The OXTR is a 389 amino acid protein encoded by a gene on chromosome 3p25. OXTR belongs to the seven-transmembrane G-protein coupled receptors (GPCR) superfamily. It shares structural similarities to the vasopressin receptor (30, 31).Many additional studies have shown that oxytocin plays important roles in numerous peripheral and central processes, including the regulation of water balance and blood osmolality (32), cardiac function (33), hunger and fat metabolism (34), bone density (35), social behavior (36) and cognitive function (37). These results are expanding the therapeutic applications of oxytocin well beyond the confines of obstetrics and gynecology.

## Sources/Origin

The brain concentration of oxytocin exceeds the peripheral concentration by approximately 1000-fold (38). Like vasopressin, oxytocin is mainly synthesized in large magnocellular neurons located in the hypothalamic paraventricular and supraoptic nuclei (38, 39). The axons reach the posterior pituitary *via* the infundibulum and the peptides are stored in vesicles that release their products into the circulation when action potentials reach the nerve endings (39). Some vasopressin is released into the median eminence where it travels *via* the hypophyseal portal system to the anterior pituitary, to simulate ACTH release in tandem with corticotropin-releasing hormone (40).

Over the years, many non-neuronal sources of oxytocin have been reported. Oxytocin may play a paracrine role in the periphery. Female reproductive tract organs such as the oviduct and uterus contain oxytocin-producing cells as do the placenta, corpus luteum, epididymis, Leydig cells, prostate, adrenal medulla, retina, thymus, and pancreas (41).

## Anti-Inflammatory Effects of Oxytocin

**Figure 1:**
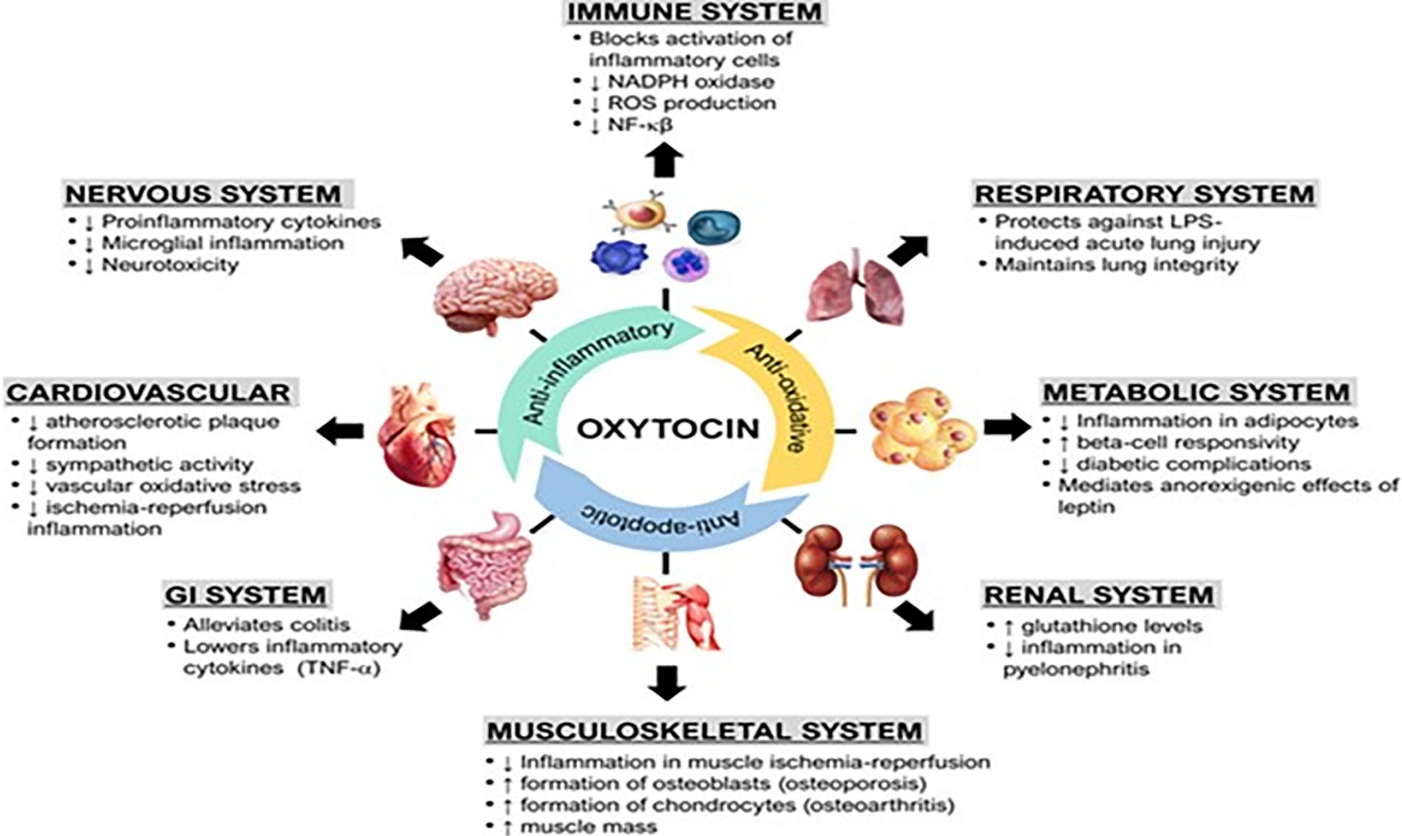
(Original by authors of the manuscript): The anti-inflammatory effects of oxytocin on various body organ systems. NADPH, nicotinamide adenine dinucleotide phosphate; NF-kB, nuclear factor kappa-light-chain-enhancer of activated B cells; ROS, reactive oxygen species; TNF-α, tumor necrosis factor-alpha. Up arrow (↑) symbol indicates increase, whereas down (↓) arrow symbol indicates decrease in the effect mentioned.

The anti-inflammatory effects of oxytocin have been reported with animal studies of multiple organ systems, including the cardiovascular, gastrointestinal, endocrine, genitourinary, and central nervous systems. Treatment with oxytocin attenuates inflammation and reduces the severity of many conditions ranging from life-threatening sepsis to complex systemic pathologies such as inflammatory bowel disease, obesity, atherosclerosis, ischemia-reperfusion injuries, pyelonephritis, osteoarthritis, and cerebral ischemia (**Figure 1** and Legend).

### Immune System

Oxytocin receptors have been detected on numerous types of immune cells including neutrophils, macrophages, and lymphocytes, and may play a significant role in immunological surveillance, defense, and homeostasis (42). Oxytocin appears to improve survival in models of sepsis. In mice, increased endogenous oxytocin levels early in the course of sepsis reduced innate immune cell activation by decreasing pro-inflammatory cytokines (such as interleukin-1 and tumor necrosis factor-alpha) (43). Oxytocin was demonstrated to suppress toll-like receptor 4 (TLR4) expression in rats (44). Oxytocin also prevents activation of free radical-damaging cascades by promoting nitric oxide (NO) release, which indirectly inhibits neutrophil leukocyte adherence and aggregation (45). Thus, oxytocin blocks neutrophils from infiltrating diverse tissues and decreases their activation as measured by myeloperoxidase activity (46, 47). Oxytocin also reduces IL-6 release and increases prostacyclin release, inhibiting platelet aggregation (46, 48). By increasing platelet and endothelial cell nitric oxide synthase (eNOS) activity, oxytocin may reverse sepsis-induced neutrophil-endothelial cell contacts and may play a role in septic shock microvascular patency (49).

There is an increase in oxytocin receptor gene expression in macrophages during inflammation, which is mediated by nuclear factor kappa-light-chain-enhancer (NF-κB) (7). NF-κB is a family of transcription factors that control immune cells (50). Oxytocin may prevent macrophages from transforming into active inflammatory cells by boosting the expression of beta-arrestin 2 (macrophage polarization), a multifunctional scaffold that modulates G protein-coupled receptors (GPCR). Along with suppression of NF-κB signaling in LPS-stimulated macrophages, oxytocin also stimulates phosphorylation (or activation) of signal transducer and activator of transcription (STAT) 6 (8). As a result, the release of TNF-alpha and other pro-inflammatory cytokines from macrophages is significantly inhibited by ligation of oxytocin to oxytocin receptor (9).

**Figure 2:**
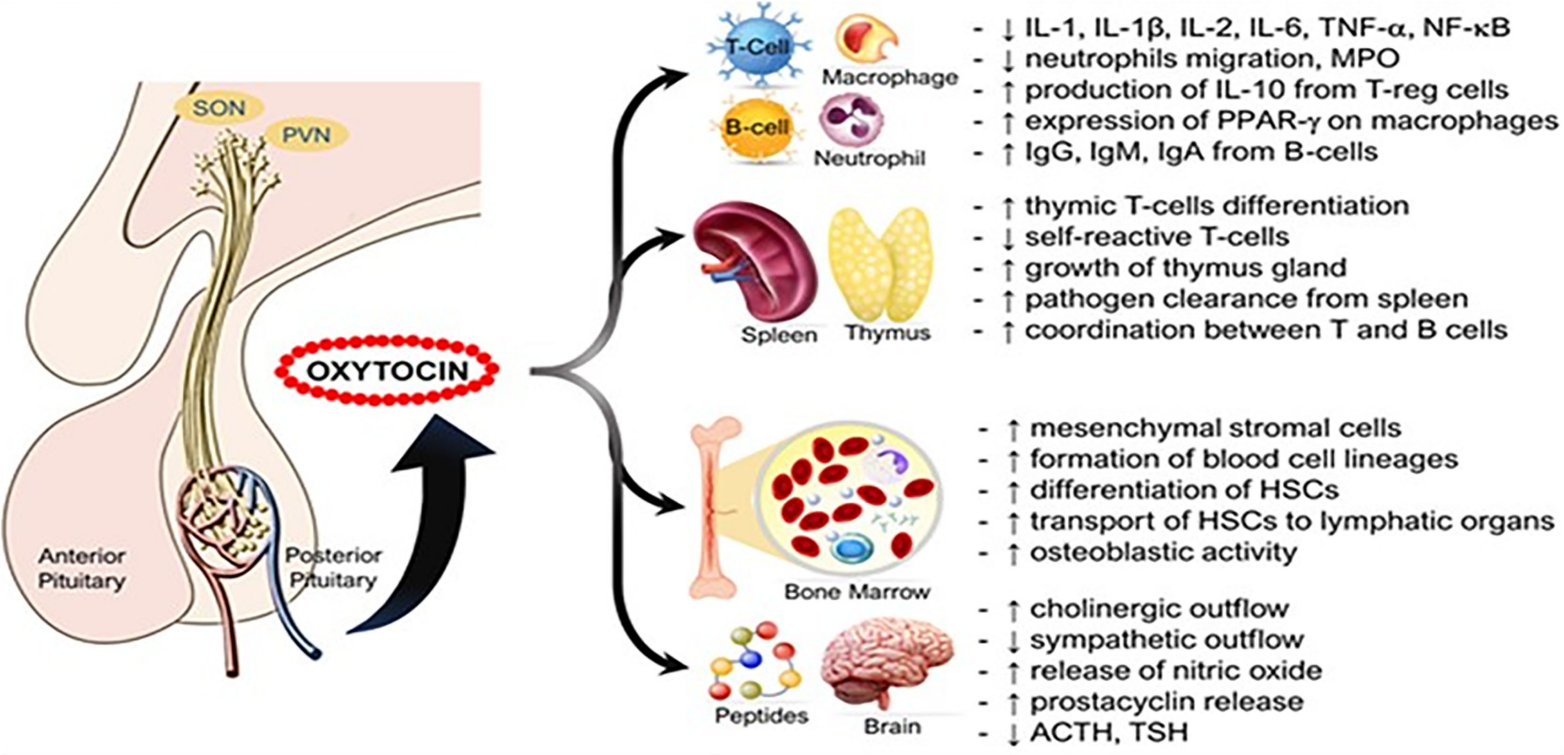
Immune functions of oxytocin. Oxytocin acts peripherally on immune cells, immune organs (thymus, spleen, bone marrow), and centrally on the nervous system to modulate its immunogenic effects. ACTH, adrenocorticotropic hormone; HSCs, hematopoietic stem cells; Ig, Immunoglobulin; IL, interleukin; MPO, myeloperoxidase; PPAR-γ, peroxisome proliferator-activated receptor gamma; PVN, paraventricular nucleus; SON, supraoptic nucleus; TSH, thyroid-stimulating hormone. Other annotations are the same as Figure 1 [Figure originates and adapted from articles (57–59)]. Up arrow (↑) symbol indicates increase, whereas down (↓) arrow symbol indicates decrease in the effect mentioned.

Oxytocin has been shown to upregulate the expression of peroxisome proliferator-activated receptor gamma (PPAR-gamma), a potent transcription factor that dampens inflammatory responses in macrophages (34, 49, 51). This suggests that oxytocin may act through PPAR-gamma to modulate inflammation (9). Oxytocin may also decrease expression of nicotinamide adenine dinucleotide phosphate (NADPH) oxidase and p38 mitogen-activated protein kinase (MAPK) to exert anti-inflammatory effects (52, 53). Oxytocin is involved in the development of self-tolerance by T cells. Oxytocin receptors (OXTRs) are found on pre-T cells in the thymus and ligation with oxytocin modulates lymphocyte maturation, differentiation, and survival (54). When lymphocytes are exposed to antigens, oxytocin stimulates their proliferation by increasing IL-2R (CD25 chain) and activation marker CD95 expression (55). In a rat model of corticosterone-induced stress, oxytocin treatment significantly reduced DNA damage in lymphocytes, likely attributed to its antioxidative capacity (56) (**Figure 2** and Legend).

### Cardiovascular System

**Figure 3:**
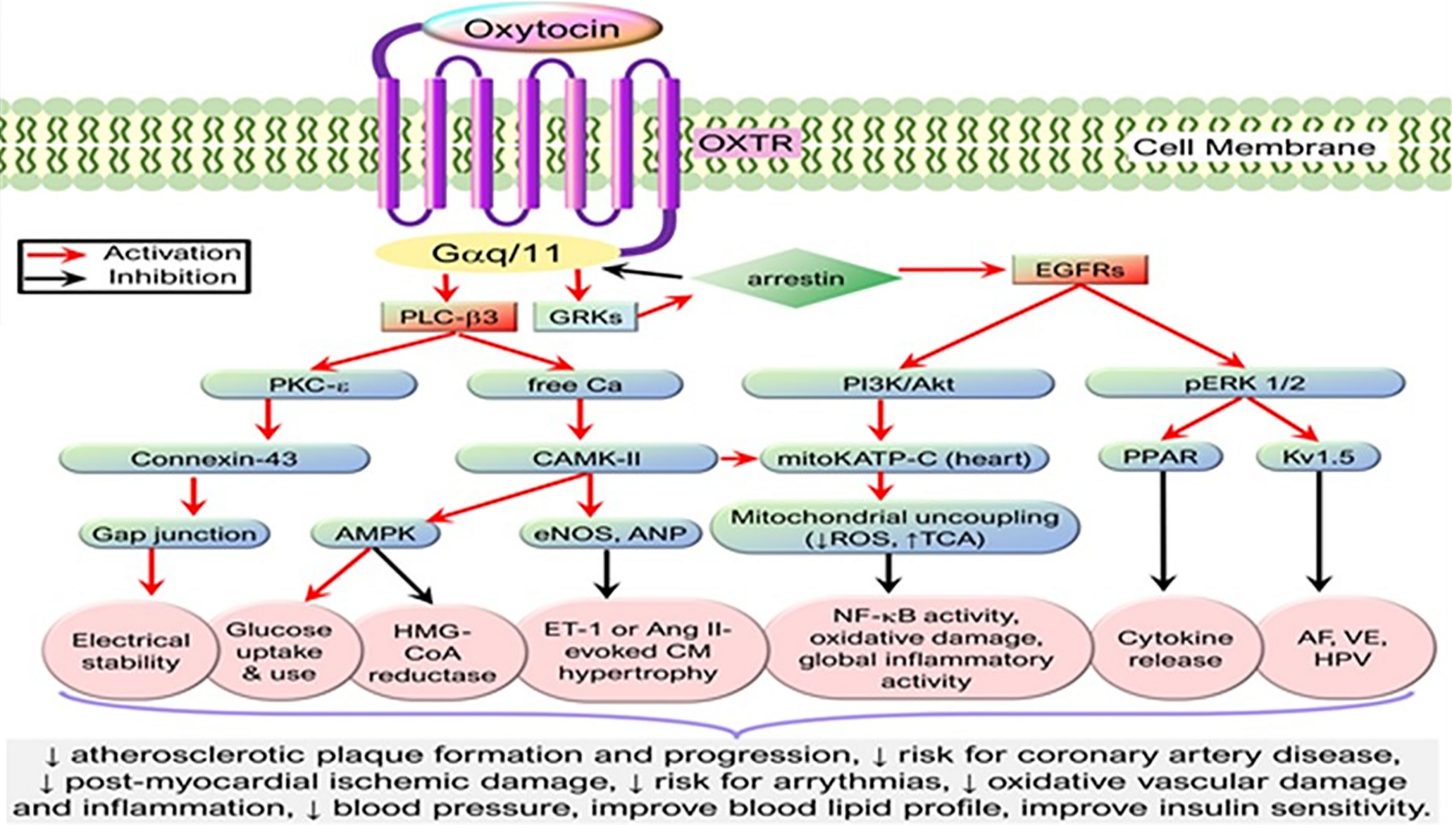
Simplified cardioprotective signaling pathways of oxytocin. Oxytocin *via* its G-protein coupled receptors triggers the PLC-β3 pathway and GRKs that cross-activate EGFR while activating their downstream signaling molecules, resulting in the activation of CAMK-AMPK, PKC-ϵ, PI3K/Akt, and pERK1/2 signaling cascades. These signaling cascades mediate cardioprotective functions such as stabilizing electrical activity, alleviating metabolic derangements, and inhibiting inflammation or oxidative stress. AF, atrial fibrillation; Akt, protein kinase B; AMPK, AMP-activated protein kinase; ANP, atrial natriuretic peptide; Ang, Angiotensin; CaMK, Ca2+/calmodulin-dependent protein kinase; EGFR, epidermal growth factor receptor; eNOS, endothelial nitric oxide synthase; ET-1, endothelin-1; GRK, G protein-coupled receptor kinase; HMG-CoA reductase, 3-hydroxy-3-methyl-glutaryl-coenzyme A reductase; HPV, hypoxic pulmonary vasoconstriction; Kv1.5, voltage-gated potassium channel Kv1.5; mKATP C, mitochondrial ATP-dependent potassium channels; OXTR, Oxytocin receptor; PI3K, phosphatidylinositol 3-kinases; PLC-β3, phospholipase C-β3; PKC, protein kinase C; TCA, tricarboxylic acid cycle; VE, ventricular ectopy. [Figure originates and adapted from articles (61–64)]. The down arrow symbol (↓) refers to decrease i.e. decrease in the pathology mentioned.

The heart and the blood vessels also synthesize oxytocin where its anti-inflammatory actions are mediated by oxytocin receptors (60) (**Figure 3** and Legend). Reduced expression of oxytocin mRNA is associated with endoplasmic reticulum (ER)-stress (65) which plays a key role in cardiovascular diseases such as atherosclerosis (66), arrhythmias (67), ventricular remodeling (68), cardiomyopathies (69), and ischemic injuries (70). Similarly, downregulation of cardiac oxytocin receptors has been linked to myocardial ischemia-reperfusion injury (71, 72). We hypothesize that a decrease in oxytocin release or oxytocin receptor numbers promotes the development of cardiovascular diseases by (a) loss of inhibition of HMG-CoA reductase enzyme and therefore increased formation of cholesterol plaques in arterial walls (61, 73). (b) The pathogenesis of atherosclerosis leading to ischemic heart disease is caused by loss of attenuation of vascular oxidative stress and increase in inflammation (74–77).

There was improved survival following myocardial infarction in mice with high plasma oxytocin levels (62). Oxytocin activates mitochondrial ATP-dependent potassium (mKATP) channels and reduces the size of myocardial infarcts, as suggested by low creatine kinase-MB isoenzyme levels, and increases in ejection fraction (78, 79). Oxytocin exerts anti-inflammatory actions in the myocardium by decreasing infiltration of immune cells (e.g., macrophages), and lowering the production of pro-inflammatory cytokines (e.g., tumor necrosis factor-alpha, interleukin-6, interleukin-1beta) into infarcted myocardium. This improves structural changes in the myocardium observed four weeks following myocardial infarctions (80). Oxytocin’s effects on atrial natriuretic peptide and nitric oxide result in negative chronotropic and inotropic effects, increased diuresis and natriuresis, decreased preload and afterload, reduced oxygen consumption, and increased cardiac output as seen in ischemia-reperfusion injury (61, 78, 81–84). In response to oxytocin injection, cardiac progenitor cells develop into mature cardiac cells and stimulate mesenchymal stem cell function in tissue regeneration following ischemia or reperfusion damage in experimental mice (81, 85).

The risk of coronary artery diseases increases significantly after menopause. The postmenopausal reductions in both oxytocin and its receptor may increase the risk of inflammation and oxidative injuries in the cardiovascular system (61, 86). Estrogen administration increases oxytocin secretion in rodents (87) and women (88), as well as the expression of oxytocin receptors in the mouse brain (87) and intracardiac oxytocin receptor signaling (89). The cardioprotective effects of estrogen may be partly mediated both by oxytocin receptor signaling and enhanced estrogen secretion.

### Central Nervous System

Oxytocin-producing neurons project to many brain areas, including the cortex, brainstem, and limbic regions. Neuroinflammation is associated with the development of cognitive defects and the progression of many neurodegenerative diseases such as Parkinson’s disease and Alzheimer’s disease (90–92). The microglia, macrophage-like immune cells in the CNS, play a major role in mediating neurotoxicity induced by inflammation (90). The major inflammatory cytokines like IL-6, tumor necrosis factor-α (TNF-α), as well as reactive oxygen species are activated by bacterial endotoxin (LPS or lipopolysaccharide) (93), β-amyloid, and interferon (IFN)-γ, which cause neuronal damage and ultimately neurodegenerative disease progression (88). Oxytocin alleviates microglia-associated neuroinflammation (94, 95).

As opposed to the pro-inflammatory properties of IL-6 in the periphery, central IL-6 may be neuroprotective in the context of cerebral ischemia (96). Oxytocin administration increased levels of central IL-6 mRNA as compared to the oxytocin antagonist (OTA) therapy. Moreover, in isolated mice, oxytocin treatments dose-dependently decreased the sizes of infarcts as compared to artificial cerebrospinal fluid-treated mice, while an oxytocin antagonist increased infarct sizes (97).

In mice, oxytocin decreased tissue loss and inflammation and also increased the expression of the antioxidative enzyme glutathione peroxidase after middle cerebral artery occlusion (98). Similarly, the neuroprotective effects of nursing on cerebral ischemia can be simulated with exogenous oxytocin supplementation in mice, decreasing apoptotic neuron death and reactive oxygen species production (99). Overall, these findings support the neuroprotective effects of oxytocin in cerebral ischemia. Oxytocin and OXTR signaling have significant effects on neurogenesis and plasticity (98) required for recovery after CNS injury (100, 101). Neurogenesis increases in the hippocampus upon oxytocin stimulation whereas inactivation of the OXTR impedes cell survival (102).

An ancient function of oxytocin is to protect against painful stimuli, including a behavioral response that alleviates stress. In mammals, endogenous oxytocin in the spinal cord regulates the processing of nociception, while in the amygdala it controls emotional pain, and regulates acute inflammatory pain in the paraventricular nucleus (103, 104).

Recent data suggest that carbetocin, a long-acting oxytocin receptor agonist, has anti-inflammatory effects *in vitro* and *in vivo*. As evidenced by fetal ultrasound, the anti-inflammatory effects of carbetocin protect against the destructive effects of interleukin-1beta (IL-1β) and low protein diet (LPD) on myelination in the double hit rat fetus model of perinatal brain injury due to prematurity plus fetal growth restriction induced by administration of a low protein diet (LPD) during gestation and potentiated by postnatal injections of subliminal doses of IL-1β (105).

Oxytocin production and secretion are vital for early development of the brain. Bakos et al, conducted a study to determine the long-term effects of neonatal oxytocin manipulation on changes in markers of brain plasticity and whether oxytocin administration protects against inflammation. Results showed developmental and sex-dependent changes in levels of nestin, microtubule- associated protein-2 (MAP-2), brain derived neurotrophic factor (BDNF) and nerve growth factor (NGF) in brains of rats, which are markers of brain plasticity. The same study also indicated that oxytocin may have a protective effect against inflammation, particularly in female rats (106).

A case report presents successful recovery of a pregnant woman who had a stroke after 34 weeks of gestation and then underwent cesarean delivery of her baby. The authors propose that oxytocin administered during and after delivery may have improved her recovery (98, 107).

In an interventional clinical trial with 23 participants having behavioral variant frontotemporal dementia (bvFTD), short-term intranasal supplementation of oxytocin was associated with satisfactory caregiver ratings and improved behavioral symptoms (e.g., levels of apathy, and expressions of empathy). The primary outcome of this phase 1 trial was safety and tolerability of 24 international units (IU), 48 IU and 72 IU of intranasal oxytocin administered twice daily for 1 week in patients with FTD as assessed by the number of participants with adverse events. Adverse events were assessed by a standardized questionnaire and monitoring of serum sodium levels (108, 109). Also, a phase 2 clinical trial with 122 participants testing intranasal oxytocin in FTD is ongoing, with assessing changes in neuropsychiatric inventory (NPI) apathy/indifference domain scores as the primary endpoint (110).

Oxytocin has been shown to exhibit a significant role in learning and memory (111). 3-Nitropropionic acid (3-NP) is a mitochondrial toxin, used to generate a Huntington’s disease (HD)-like model (112). In an experiment on 3-NP injected rats, OXT improved memory and learning performance in both male and female rats (113). However, intracerebroventricular (ICV) administration of OXT reversed defeat-induced social avoidance only in male rats (114). A recent controlled clinical trial consisting of 9 non-medicated males with CAG-expansion HD and 10 age-matched healthy controls provide evidence that intranasal oxytocin normalizes abnormal brain activity measured by functional MRI (fMRI) in the HD patients to levels similar to controls. An emotional face matching task was tested and the left middle frontal gyrus and left putamen areas visualized by means of fMRI (115).

Recently, many *in vivo* and *in vitro* studies have demonstrated an effect of oxytocin on adult neurogenesis in rats and is postulated to stimulate neuronal growth in the hippocampus of rats (116). Administration of 1-methyl-4-phenyl-1,2,3,6-tetrahydropyridine (MPTP), a neurotoxin, induces symptoms of Parkinson’s disease in mice. Oxytocin levels are decreased in these animals. Supplementation with exogenous oxytocin effectively rescues the locomotor disabilities and anxiety-like behaviors, as well as loss of dopaminergic neurons and oxidative stress in MPTP mice. Oxytocin treatment appears to activate the miR-26a/DAPK1 signaling pathway (117, 118).

### Gastrointestinal Diseases

Previously assumed to be a hormone with a minor effect on digestion, recent research reveals a more significant role for oxytocin in the digestive tract. There is evidence now that oxytocin receptors are expressed on both circular and longitudinal muscle cells of the stomach (119), intestinal mucosa, and enteric nervous system (120), but the effects of oxytocin on gastrointestinal motility are species-specific. For instance, oxytocin acts directly on the human stomach to accelerate gastric emptying (121, 122), but acts indirectly *via* cholecystokinin to delay gastric emptying in rats (123, 124). Also, oxytocin improves antegrade colonic motility (125) that is associated with intestinal disorders (126). Moreover, intranasal oxytocin relieves abdominal pain, discomfort, and depression associated with irritable bowel syndrome (127, 128).

**Figure 4:**
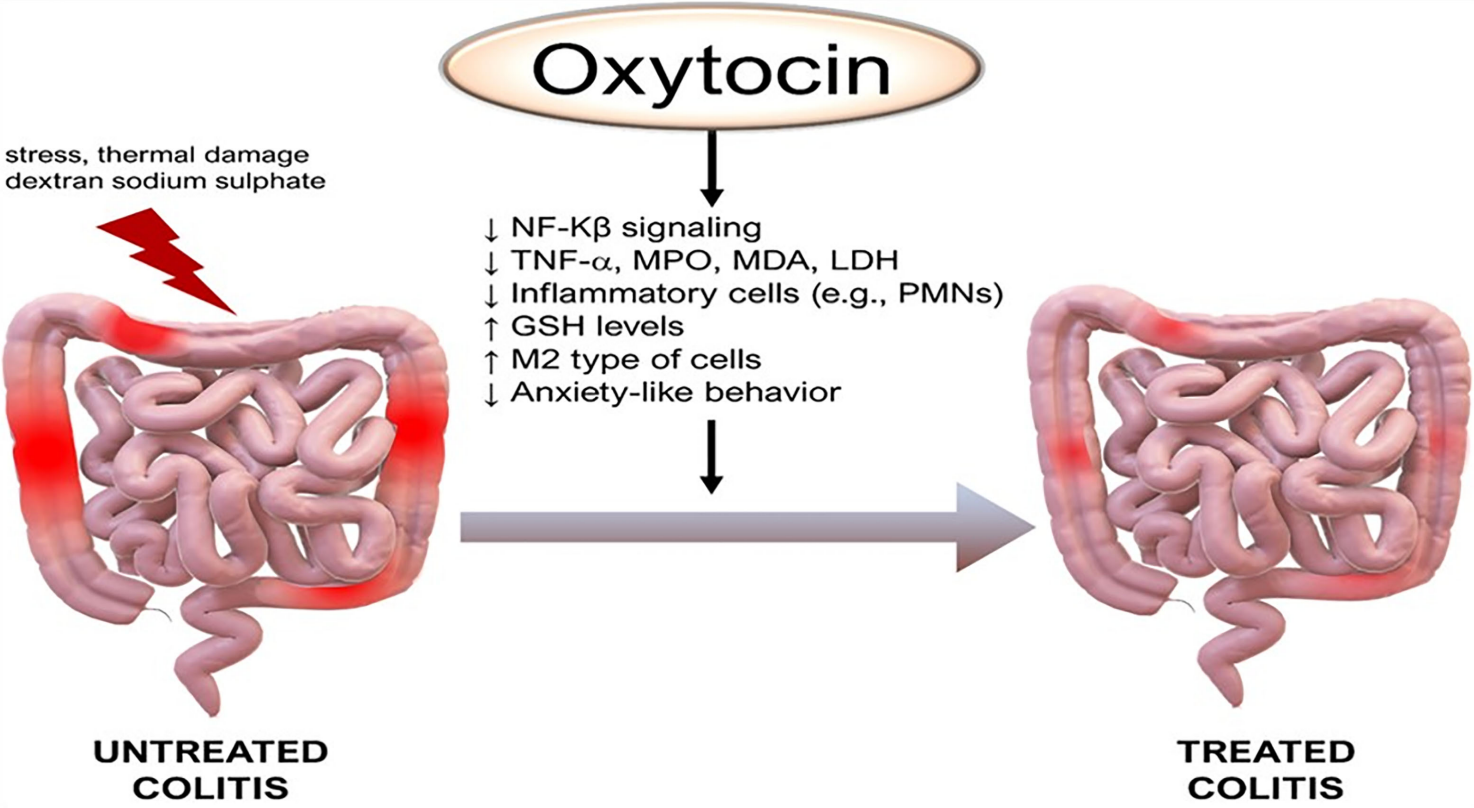
Anti-inflammatory effects of oxytocin on the colon. Of note, M2 are anti-inflammatory macrophages that contribute to tissue healing. GSH, glutathione; LDH, lactate dehydrogenase; MDA, malondialdehyde, PMNs, polymorphonuclear leukocytes. Other annotations are the same as **Figure 1**. (Original by the authors of the manuscript). Up arrow (↑) symbol indicates increase, whereas down (↓) arrow symbol indicates decrease in the effect mentioned.

In a mouse models of colitis induced by either dextran sulfate sodium (DSS) or 2,4,6-trinitrobenzene sulfonic acid (TNBS), disease severity was increased in oxytocin receptor (OXTR) deficient mice when compared to wild-type controls. In addition, oxytocin treatment reduced the severity of colitis in mice (129). In another mouse study, oxytocin attenuated acetic acid-induced colitis by decreasing serum tumor necrosis factor-alpha and lactate dehydrogenase, reducing colonic malondialdehyde and myeloperoxidase levels, and restoring colonic glutathione levels (130). It was recently reported that oxytocin decreased the sensitivity of macrophages to lipopolysaccharide stimulation with lower expression of inflammatory cytokines, like IL-1β, IL-6, and TNF-α, but increased the sensitivity to IL-4 stimulation with enhanced expression of M2-type genes, arginase I (Arg1), CD206, and chitinase-like 3 (Chil3). This bidirectional modulation was partly due to the up-regulation of β-arrestin2 and resulted in the inhibition of NF-κB signaling and promotion of STAT 6 phosphorylation (7). These findings support a role for oxytocin in treating a number of gastrointestinal disorders such as gastroparesis, gastropathy, irritable bowel syndrome, and inflammatory bowel disease (**Figure 4** and legend).

### Metabolic Diseases

In addition to immune-modulating functions, oxytocin affects many metabolic diseases. A study with mice showed that oxytocin receptor deficiency is associated with the development of late-onset obesity (131). Similarly, in a human study, low plasma oxytocin levels have been reported in obese groups, new-onset type 2 diabetes, and hyperlipidemia (132). Administration of oxytocin, either centrally or peripherally, mediates the anorexigenic actions of leptin, reduces food intake, and lowers weight gain as shown in multiple mice models (133–137). Oxytocin also reduces chronic inflammation in obese mice (138, 139). Ironically, oxytocin is less effective in enhancing labor in obese women (140–142).

**Figure 5:**
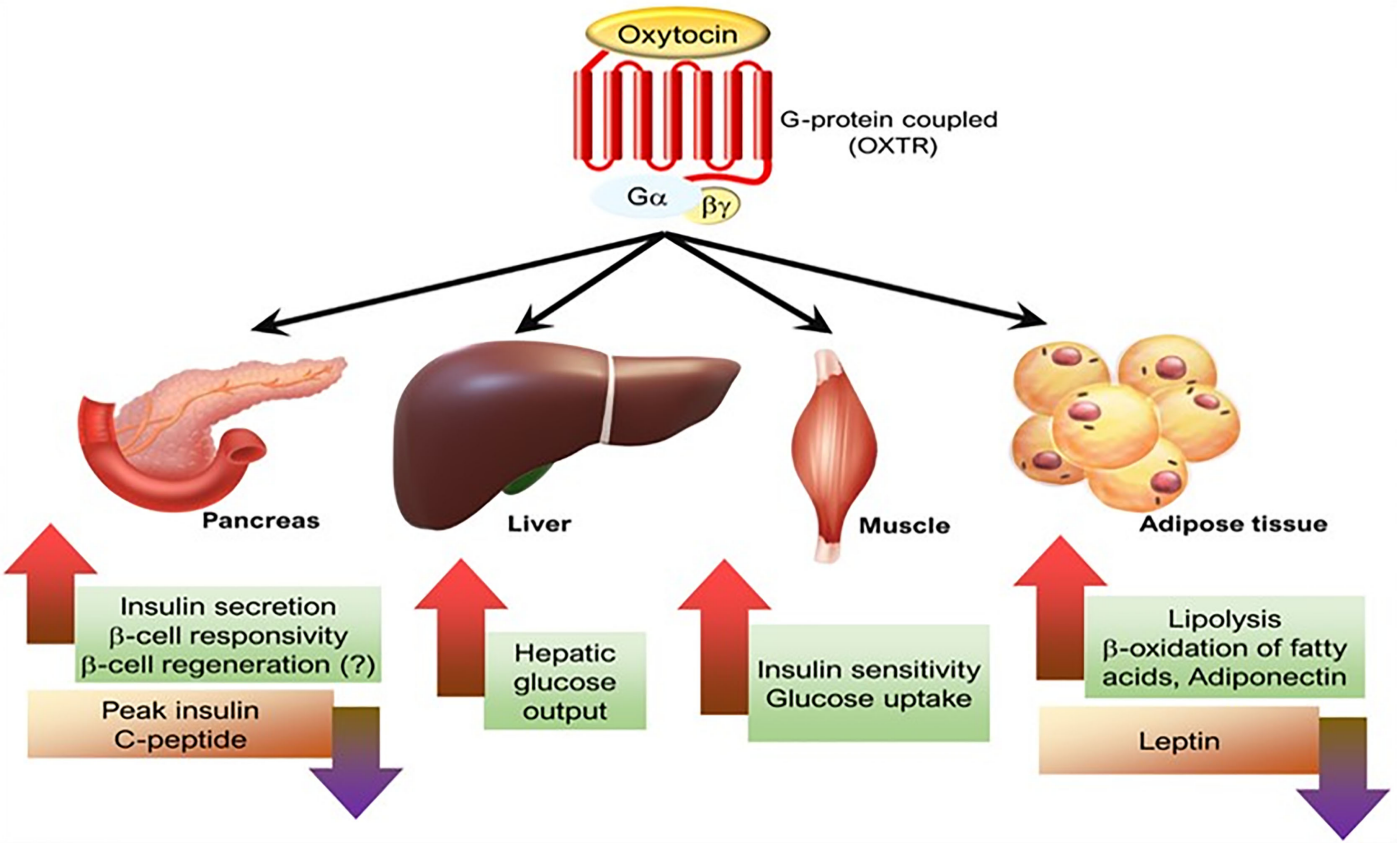
The effects of oxytocin on various metabolic organs. [Figure originates and adapted from article (149)].

Studies in rodents and humans have shown that systemic administration of oxytocin affects lipid metabolism and, secondarily, other related conditions. It lowers blood pressure, attenuates the hyperglycemic state in diabetes, improves insulin secretion and sensitivity, and prevents diabetic complications (e.g., diabetic cardiomyopathy, diabetic osteopathy, retinopathy) (63, 143–148). We look forward to studies of the therapeutic potential of oxytocin in the treatment of various metabolic conditions such as obesity, hypertension, and diabetes (**Figure 5** and legend).

### Dermatological System

The healing of wounds is impacted by many factors including psychological stress (150). Multiple studies of animal models have shown that exogenous treatment with oxytocin speeds wound healing (151, 152). One experimental study demonstrated that administration of Lactobacillus reuteri bacilli in drinking water upregulates the expression of oxytocin in the hypothalamus and accelerates wound epithelialization in mice (153). In a clinical trial on patients with diabetic foot ulcers, intra-arterial injection or local application of oxytocin results stimulates vasculogenesis, (the proliferation of endotheliocytes and histiocytes) which, in turn, promotes effective wound clearance and formation of optimal granulation) (154).

Oxytocin has a preventive impact on tissue damage owing to its anti-inflammatory and antioxidant properties, but its effect on wound healing is not restricted to that. The potential of oxytocin, by means of its anti-apoptotic and angiogenic properties, optimizes the activity of mesenchymal stem cells to improve tissue healing in rats (155–158). Oxytocin has been demonstrated to influence plasma levels of both insulin growth factor-1 (IGF-1) and growth hormone (GH), which contributes to its immune-modulatory and regenerative effects (155). Oxytocin also protects human skin cells from oxidative damage (159).

### Renal system

The role of oxytocin in renal hemodynamics was proposed in a rat model when oxytocin binding sites were initially recognized in the renal cortex, medulla, and macula densa (160, 161). Identifying oxytocin receptors both in the cortex and medulla of embryonic and developing kidneys suggests that oxytocin may play a role in both renal development and renal function (162).

Oxytocin participates in osmoregulation. For instance, administration of oxytocin to dehydrated female Wistar rats increased endogenous vasopressin. Whereas when ‘water load’ is simulated by administering 5ml of water per 100gm body weight through a gastric probe decreased vasopressin levels and increased endogenous oxytocin levels supporting oxytocin’s role in diuresis following hyperhydration. When exogenous oxytocin is administered in water load or hyperhydration, the diuretic effect is optimized, confirming the role of oxytocin in diuresis (163). Another experiment was conducted using three oxytocin antagonists to test their effects on oxytocin and vasopressin on osmoregulation. The results revealed that the oxytocin antagonists significantly decreased oxytocin’s ability to increase urine production and sodium excretion. While they had no effects on vasopressin’s anti-diuretic or natriuretic actions. These results suggest that different receptors may be involved in the renal effects of both peptides (164).

The application of exogenous oxytocin has been shown to protect the renal system against the oxidative stress seen acute renal failure and against nephrotoxins. For instance, cisplatin-induced renal injury involves activation of nuclear factor-kappa B (NF-κB) and over-expression of tumor necrosis factor-alpha (TNF-α), transforming growth factor-beta (TGF-β), monocyte chemo-attractant protein-1 (MCP-1), intercellular adhesion molecule (ICAM), heme oxygenase-1, and TNF receptor 1 (TNFR1) and TNF receptor 2 (TNFR2). These cytokines and receptors cause infiltration of lymphocytes and macrophages into the renal medullary interstitial zone resulting in inflammation, fibrosis, and impaired renal function (52, 165). Studies using mouse models of cisplatin-induced renal injury show that oxytocin treatment lowers the gene expression of NADPH oxidase, inhibits p38 mitogen-activated protein kinases (p38 MAPK), activates Akt signaling pathway, and decreases the renal overexpression of transforming growth factor-beta1 and C-reactive protein. In addition, oxytocin administration decreases renal production of superoxide anion (O2-) and proinflammatory cytokines that further reduces infiltration of inflammatory cells such as macrophages into the renal interstitium and improves the histological appearance of the renal tubules. The anti-oxidative effects also include increases in renal glutathione and superoxide dismutase levels (166). Together, these anti-inflammatory and anti-oxidant effects of oxytocin significantly protect against cisplatin-induced renal injury.

Like nephrotoxins, the pathogenesis of infectious agents includes inflammation during urinary tract infections such as pyelonephritis. A study of the effects of oxytocin treatment on *Escherichia coli*-induced acute and chronic pyelonephritis demonstrated that serum LDH, TNF-alpha, and creatinine decreased significantly after treatment with oxytocin 24 hours and one week after the infection began. Oxytocin also lowered myeloperoxidase activity, indicating that its protective effects might be mediated primarily by inhibition of release of reactive oxygen species and inflammatory cytokines by neutrophils. Infection- induced oxidative stress resulted in low levels of renal glutathione that was blocked by administering oxytocin. Histopathological improvements in glomerular structures and reduced inflammation after infection support the anti-inflammatory effects of oxytocin on the renal parenchyma in preclinical models of pyelonephritis (45).

### Effect of Oxytocin on Bone

Osteoporosis is a major health care issue worldwide. Oxytocin contributes to the regulation of bone mass by promoting the formation of osteoblasts and is emerging as a potential treatment option for osteoporosis (167). Oxytocin receptors are expressed by osteoblasts and osteoclasts. Oxytocin induces the proliferation of human osteoblast-like cells *in vitro* (58).

Several studies have shown that activation of the oxytocin receptor (OTR) increases intracellular calcium levels in osteoblasts (168). This increase in calcium activates the extracellular signal-regulated kinase 1/2 (ERK1/2) pathway which leads to phosphorylation of core binding factor subunit alpha-1 (CBFA-1), a transcription factor essential for osteoblast differentiation, also known as runt-related transcription factor 2 (RUNX2), which increases osteogenesis (169).

Mice lacking oxytocin or oxytocin receptors exhibit severe osteopenia which can be normalized by oxytocin administration (170). Oxytocin reduced osteopenia in the femoral neck of periestropause rats, suggesting its possible use for preventing primary osteoporosis (171).

Like osteoporosis, osteoarthritis (OA) is a multifactorial disorder. Oxytocin has shown to have effect on chondrogenesis and osteoarthritis. A cohort of 63 men and 19 women with hand OA were studied. There was a significant association between osteoarthritis and low circulating levels of oxytocin. The stimulatory effect of oxytocin on chondrogenesis was shown to play a role in the pathophysiology of osteoarthritis (172). In addition, oxytocin has been administered *via* intrathecal injection for treating lower back pain due to its analgesic action (173).

## Effects of Oxytocin in Sepsis

As discussed above, oxytocin has emerged as a potent anti-inflammatory and anti-oxidant peptide in the last few decades. This led researchers to study the effects of oxytocin in preclinical models of sepsis. Various organ systems (cardiovascular, nervous, and respiratory systems) have been examined. The results of the experiments described below are encouraging and strongly warrant future research with oxytocin in the setting of sepsis.

### Cardiovascular System

During sepsis, elevated cytokines (tumor necrosis factor-alpha, interleukin-6, interleukin 1 beta) alters sympathovagal balance (174, 175). This results in decreased heart rate variability (HRV), changes in heart rate, and conduction delays (176–178). Reduced HRV has been associated with unfavorable outcomes in sepsis (179, 180). In septic rodents, treatment with exogenous oxytocin restores the normal heart rate values, and favors cardiac pacemaker coupling with cholinergic influences. This has been evident on telemetric electrocardiogram through improvement in two parameters of HRV i.e., high frequency heart rate and low frequency heart rate (181, 182). This suggests that oxytocin modulates the cardiac autonomic nervous system during sepsis.

Sepsis-induced myocardial dysfunction, also known as septic cardiomyopathy, is a feature of severe sepsis (183). Pro-inflammatory cytokines and oxidative stress play a central role in septic cardiomyopathy (184, 185). The pathophysiology of sepsis-induced myocardial dysfunction is not fully understood, but a disease-associated reduction in tissue cystathionine-γ-lyase (CSE) expression, an endogenous H2S-producing enzyme associated with impaired organ function, has been reported. CSE-mediated cardio-protection has been suggested to be related to the upregulated expression of the oxytocin receptor (OTR). CSE can also mediate glucocorticoid receptor (GR) signaling, which is important for normal heart function. Therefore, a potential interplay between GR, CSE, and OTR in sepsis-mediated oxidative stress, inflammation and cardiac dysfunction has been postulated (186, 187). Another recent study reported that cystathionine-γ-lyase (CSE) mediates cardio protection through upregulation of oxytocin receptors *via* reperfusion injury salvage kinase (RISK) pathway (61). The administration of the slow H2S-releasing compound GYY4137 restored the native levels of cardiac oxytocin receptors. H2S has been reported to work through a VEGF-dependent cardioprotective pathway (188). Both OXTR and H2S regulate nuclear factor E2-related factor 2 (NRF2), an important antioxidant transcriptional regulator (189). In addition to its antioxidant effects, oxytocin also reduces the production of pro-inflammatory cytokines (such as tumor necrosis factor-alpha, interleukin-1 beta) found in sepsis models (46, 93). The dual anti-inflammatory and anti-oxidative effects of oxytocin make it a unique cardioprotective peptide in sepsis.

### Central Nervous System

**Figure 6:**
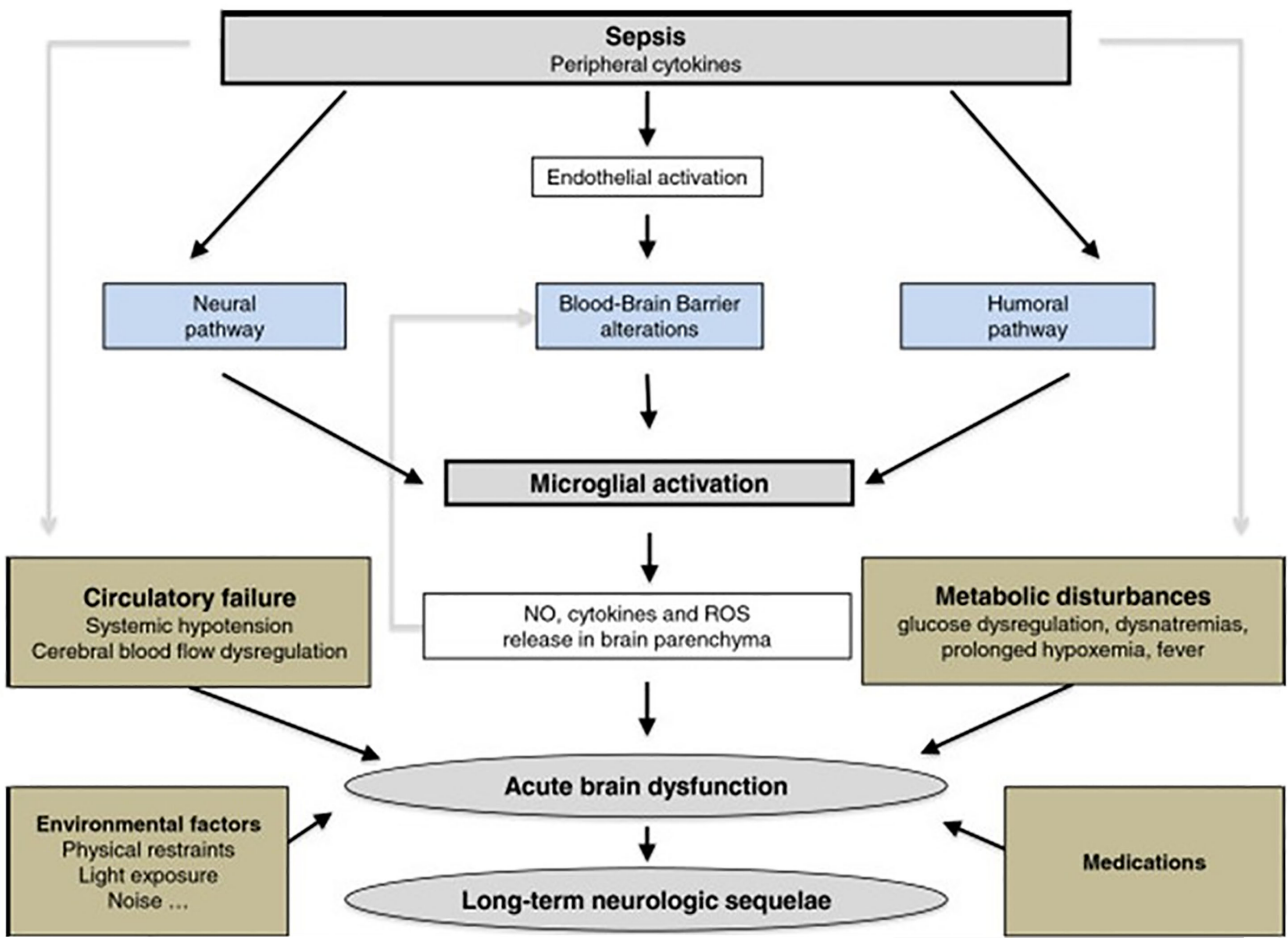
The response of the brain to systemic infection is physiologically triggered by an activating signal that is mediated by three pathways. 1) The neural pathway that requires activation of primary afferent nerves, such as the vagus or the trigeminal, by involving peripherally produced pathogen-associated molecular patterns (PAMPs) and cytokines. 2) The humoral pathway involves circulating cytokines. They reach the brain at the level of the choroid plexus and the circumventricular organs that lie outside the blood–brain barrier (BBB). 3) The blood–brain barrier alterations induced by the activation of cerebral endothelial cells result in the release of various mediators into the brain. This activation is due to the production, at an early phase of sepsis, of nitric oxide synthase-derived nitric oxide. All of these pathways instigate the activation of microglial cells, which are the resident immune cells of the brain. When activated, microglial cells may negatively affect the brain by the production of nitric oxide, cytokines, and reactive oxygen species that lead to cell death within vulnerable areas of the brain. This production is, in itself, responsible for an increase of the BBB alterations, thus causing a vicious circle of increasing brain dysfunction and injury. These mechanisms are compounded by common metabolic disturbances that occur in septic patients e.g. prolonged hyperglycemia, severe hypoxemia, hemodynamic failure, use of medications, as well as iatrogenic and environmental factors. Septic-associated brain dysfunction may be associated with neurologic sequelae in survivors, including functional and cognitive decline, probably by neurodegenerative and/or ischemic mechanisms [Figure originates and adapted from articles (194, 195)].

Sepsis induces inflammation in the central nervous system (CNS) that may cause cognitive and neuroendocrine changes (190). Brain dysfunction, including acute aberrations in vegetative or autonomic functions (191) as well as permanent alterations in sepsis survivors (192), is a hallmark of sepsis. Circulating immune cells produce peripheral inflammatory mediators that increase blood-brain barrier (BBB) permeability allowing inflammatory mediators access to the brain parenchyma and initiating neuroinflammatory processes (193). These cytokines, in association with endothelial dysfunction and reduced cerebral blood flow, contribute to impaired mitochondrial function in neurons (194), programmed cell death in the CNS, and cerebral deficits (195) during sepsis (**Figure 6** and legend).

**Figure 7:**
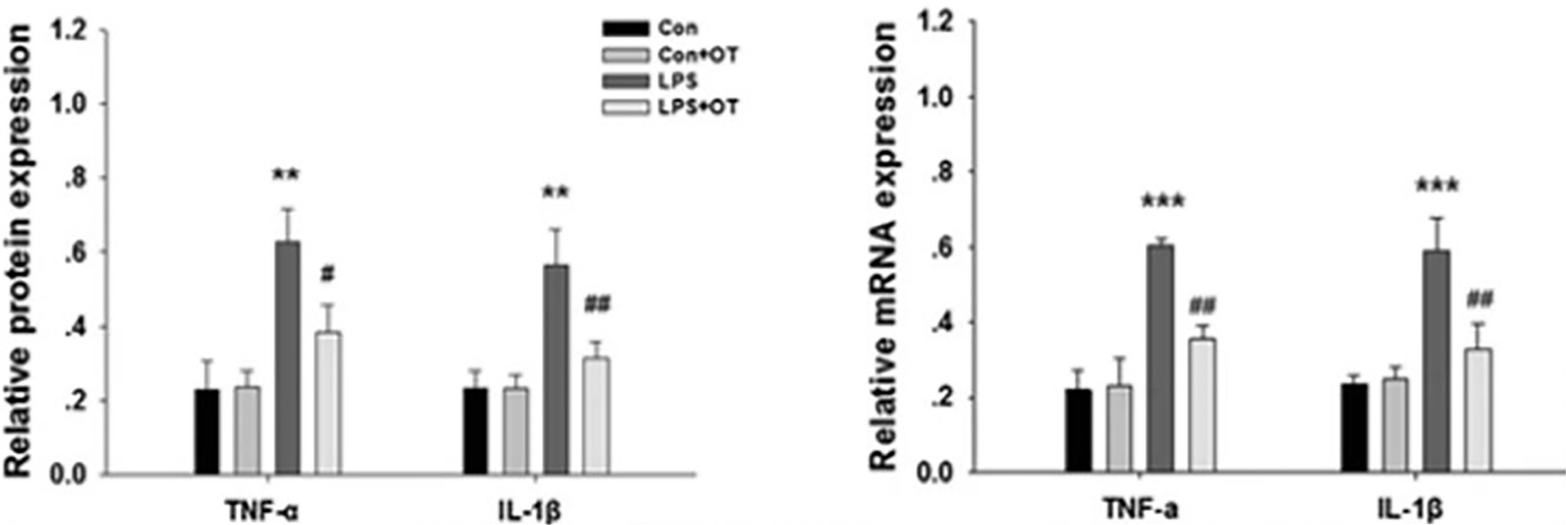
Primary microglia were pre-treated with or without OT (1 μM) for 2 h followed by LPS (500 ng/ml) for 24 h. The levels of TNF-α and IL-1β were analyzed by Western blotting. β-actin was used to evaluate protein loading. The levels of TNF-α and IL-1β mRNA were analyzed by RT-PCR, and β-actin was used to evaluate protein loading. Each value was normalized to β-actin. Values represent the mean ± S.D. of three independent experiments. **p<.01, ***p < 0.001 LPS vs Control; #p < 0.05, ##p < 0.01 LPS + OT vs LPS] [Figure from Springer Nature (90) permissible to re-use under a CC-BY 4.0 license].

Oxytocin appears to protect the brain from injury during sepsis. An increase in oxytocin and vasopressin in the initial phase of sepsis may reflect the brain’s attempt to combat the detrimental consequences of the disease (190). Oxytocin receptors are present on neurons, astrocytes, and microglia (104). Microglia play a significant role in the pathogenesis of brain dysfunction during sepsis (196). When microglia are exposed to inflammatory cytokines, they are activated and produce cytokines that further contribute to neuroinflammation during sepsis (197, 198). Oxytocin pre-treatment of LPS-stimulated BV-2 cells (microglial cells derived from C57BL/6 mice) and primary microglia significantly decreased the expression of LPS-induced IL-1β and TNF-α. Similarly, pre-treatment with oxytocin reduced the LPS-stimulated increase in cyclooxygenase (COX-2) and inducible nitric oxide synthase (iNOS) in BV-2 cells and microglial cells (90) (**Figure 7** and legend).

**Figure 8:**
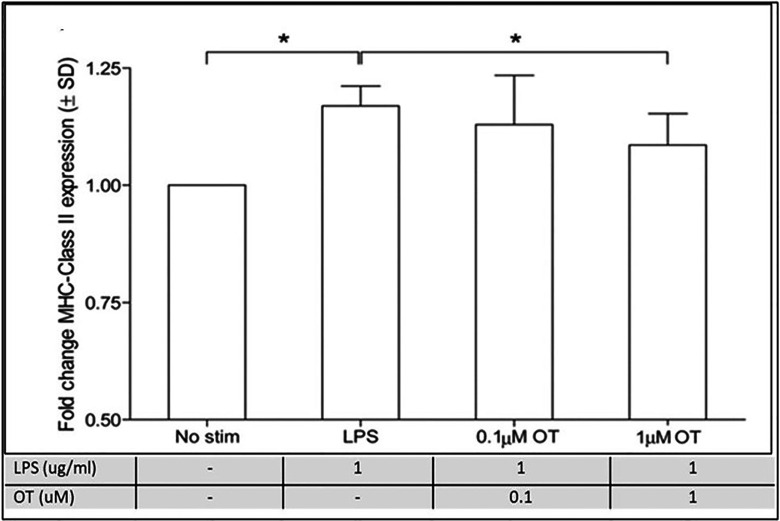
Oxytocin suppresses microglial reactivity *in vitro*. A) MHC class II expression is up-regulated following a 24-hour LPS challenge (1 μg/mL) in microglial cells. Preincubation with the high dose (1 μmol/L), but not the low dose (0.1 μmol/L), OT dose-dependently attenuates LPS-stimulated MHC class II expression. However, this effect is blocked by coincubation with oxytocin receptor antagonist (1 μmol/L), n=7 per treatment condition, data are expressed as mean ± SD. *Indicates a statistically significant difference between indicated groups, (P>0.05) (90, 97). Figure from Open access AHA journals (97) permissible to re-use under a CC-BY 4.0 license.

In another study of rats oxytocin dose-dependently reduced LPS-stimulated MHC Class II, a marker of microglial activation and reactivity (90, 97) (**Figure 8** and legend).

Sepsis can also cause acute alterations in the neuroendocrine system (199), particularly in the hypothalamus (190). Microglial activation in the hippocampus resulted in excess reactive oxygen species (ROS), cytokine formation and protracted cognitive dysfunction in sepsis survivors (200). Oxytocin administration after an LPS challenge *in vivo* not only decreased the expression of inflammatory cytokines but also the number of prefrontal cortical cells labeled with ionized calcium-binding adapter molecule 1, a microglial activation marker (104).

Important mechanisms of neurosepsis include microglial activation, altered neurotransmission, impaired cerebral perfusion, and blood-brain-barrier dysfunction (201). In mice with acute brain inflammation induced with LPS, administration of intranasal oxytocin before the LPS injection mitigates microglial activation (90, 104). Surprisingly, oxytocin has no effects in the absence of LPS administration, demonstrating that anti-inflammatory effects of oxytocin require the presence of a stressor (104). Similar results of oxytocin were reported in healthy men exposed to an LPS challenge (93). Men who received an LPS challenge along with oxytocin, had a reduced cytokine, chemokine, and neuroendocrine response compared to men who were administered LPS alone. In conclusion, these findings suggest that oxytocin can mitigate the inflammatory response stimulated by LPS in several species (104).

Modifications of microglial activation, utilization of antioxidants, and prevention of blood-brain-barrier dysfunction may represent important therapeutic targets of oxytocin to improve neurological outcomes (201).

### Respiratory System

The lung injury is common in sepsis (202). Oxytocin receptors are expressed on human airway smooth muscles (203, 204) and endothelial cells lining blood vessels including pulmonary arteries (205). It has been reported that in mice endotoxemia increases respiratory rate and decreases respiratory sinus rhythm, resulting in impaired alveolar gaseous exchange and ventilation/perfusion mismatch. Oxytocin administration normalizes the respiratory rate and improves respiratory sinus arrhythmia (206).

Additional protective effects of oxytocin have been reported to be mediated by nitric oxide (NO) (207). An increase in endothelial NO production may inhibit the adherence of neutrophils to the endothelium and prevent the formation of platelet-neutrophil aggregates in hyperinflammatory conditions such as sepsis (49). Moreover, LPS-induced acute lung injury and pulmonary edema was ameliorated by eNOS in adipose tissue-derived stem cells (208).

Studies have suggested that administration of exogenous oxytocin protects the lung from injury under conditions of chronic stress (209).

It has recently been shown that intraperitoneal injection of LPS leads to acute lung injury in experimental animals. Treatment with oxytocin attenuated lung edema and damage to lung tissue. Furthermore, oxytocin treatment decreased infiltration of leukocytes, the expression of NF-κB, and the appearance of pro-inflammatory cytokines in the lungs (210). Oxytocin has also been shown to alleviate the consequences of a heat-stroke induced acute lung injury in rats (211).

Together, these findings support the protective effects of oxytocin in protecting against sepsis-related lung injury.

### Gastrointestinal System

Sepsis is associated with an increased risk of damage to gastrointestinal organs. For instance, decreased synthesis of proteins in gastric mucosa is found in the septic mice, and this results in an increased risk of gastric stress ulcers and bleeding during sepsis (212). In Wister rats, endotoxin stimulates the oxytocin release within the central nervous system, which in turn acts on the dorsal motor nucleus of the vagus to inhibit gastric acid secretion (213). Studies in Wistar rats and guinea pigs confirmed the gastric antisecretory properties of oxytocin (214, 215). Results showed that oxytocin can significantly protect gastric mucosa from injury induced by ischemia-reperfusion *via* the oxytocin receptor. This protective effect is mediated through oxytocin’s effects on the vagus nerve and the sympathetic system by reducing gastric juice output and gastric acidity (216).

Cecal ligation and puncture-induced sepsis, a commonly accepted model of polymicrobial sepsis in rodents, is characterized by increased levels of serum tumor necrosis factor-alpha, inflammatory cell infiltration in target organs, enhanced fibrotic activity, lower glutathione levels, and elevated malondialdehyde levels in the liver and colon. Oxytocin treatment reduces serum tumor necrosis factor-alpha levels and the infiltration of neutrophils, as indicated by low myeloperoxidase oxidase levels in the liver and colon. Colonic tissue of oxytocin-treated animals exhibits decreased lipid peroxidation products, reactive oxygen species, malondialdehyde levels, and increased glutathione levels (130). Oxytocin may also inhibit the production and deposition of extracellular matrix components that contribute to hepatic fibrosis during sepsis (130). Thus, oxytocin administration may ameliorate sepsis-associated inflammatory and oxidative damage to gastrointestinal organs.

### Renal System

Oxytocin has only recently been considered as a candidate for alleviating sepsis in the renal system. Nevertheless, the findings of a limited number of studies are encouraging and emphasize the need for more extensive research in this area. The inflammatory response during sepsis damages renal cells, causing a decline in renal function and renal failure (217). The effects of oxytocin on the renal parenchyma are mediated, in part, by inhibiting pro-inflammatory cytokine production (e.g IL-1 and IL-6). This was demonstrated experimentally during renal ischemia-reperfusion injuries. Oxytocin treatment significantly improved renal function, as evidenced by decreased serum creatinine levels and blood urea nitrogen (BUN) levels, restored normal renal histopathology, and improved antioxidant status in the kidney tissues. In addition, decreases in lipid peroxidation (reduced MDA), polymorphonuclear leukocytes (PMNs) infiltration (as determined by reduced myeloperoxidase (MPO) activity) and reactive oxygen species (reduced lucigenin) were also reported (218, 219).

A study postulated that oxytocin significantly reduced sepsis-induced kidney damage. The results demonstrated that loss of the brush border, tubular dilatation, and mononuclear cell infiltration were significantly decreased in oxytocin treated rats following sepsis induced by cecal ligation and puncture compared to saline treated group (220) (**Table 1**).

**Table 1:** Results from studies by Çavuşoğlu et al. comparing the effects of oxytocin in CLP sepsis.

	Normal Group	Sham operated Group	CLP Group	CLP and saline Group	CLP and 0.4mg/kg oxytocin Group
Loss of brush border	0.1 ± 0.1	0.1 ± 0.1	2.8 ± 0.1**	2.6 ± 0.2**	1.6 ± 0.2^#^
Tubular dilation	0	0.3 ± 0.3	2.6 ± 0.3**	2.8 ± 0.1**	1.8 ± 0.3^#^
Mononuclear histological index (MNHI)	0.1 ± 0.1	0.6 ± 0.2*	2.5 ± 0.5**	2.8 ± 0.1**	1.1 ± 0.1^##^
Erythrocyte extravasation	0	0.1 ± 0.1	0.8 ± 0.1*	1.1 ± 0.1*	1.0 ± 0.2
Cast Formation	0	0.1± 0.1	0.6 ± 0.2*	0.8 ± 0.1*	1.1 ± 0.1

Results were represented as mean ± SEM. *p < 0.05, **p < 0.00001, different from normal and sham-operated groups; ^#^p < 0.05, ^##^p < 0.001, different from CLP and CLP + saline group. Comparison is done based on the levels of Plasma TNF- ∝, MDA, CRP, WBC and Creatinine levels. Results demonstrated that loss of brush border in proximal tubule cells around the glomerular structure, dilatation of proximal and distal tubules and MNHI were significantly less in CLP+ Oxytocin treated group; while cast formation in tubules, change in glomerular structure and erythrocyte extravasation were similar when compared to CLP and CLP+ saline group (220).

Table is from Suleyman Demirel University Journal of Health Science permissible to reuse under a CC-BY 4.0 license (open access).

Oxidant injury has been implicated in the pathogenesis of renal inflammatory processes, which in turn cause structural alterations, and changes in the amino acid transport system at the renal brush border. These changes lead to damage in membrane lipids, proteins and nucleic acids and ultimately apoptotic cell lysis within the kidneys (221). Oxytocin has been shown to reduce sepsis-induced lipid peroxidation as evidenced by decreased malondialdehyde (MDA) levels, serum CRP levels and renal structural damage (46, 219, 220) Table (1) The increase in serum levels of TNF-α, a key pro-inflammatory cytokine, in sepsis, is also significantly ameliorated by oxytocin treatment (220).

Thus, numerous studies support the beneficial effects of oxytocin administration in experimental models of sepsis. However, additional studies need to be conducted to better understand the potential benefits of oxytocin therapy in sepsis-related renal damage.

### Musculoskeletal System

Skeletal muscle degeneration or muscle wasting is a common finding sepsis. Sepsis exposes skeletal muscles to inflammation and oxidative stress causing sarcopenia. Oxytocin administration can counteract sarcopenia by its regenerative potential on striated muscles (222).

Similarly, another study comparing melatonin and oxytocin in the prevention of critical illness polyneuropathy rats with experimentally induced sepsis showed that individually melatonin and oxytocin or co-administration of melatonin with oxytocin abolishes the nerve electrophysiologic alterations, increases muscle strength and suppresses oxidative stress, lipid peroxidation, and TNF-α release in muscles caused by sepsis (223). Both oxytocin and melatonin have significant benefits against sepsis induced polyneuropathy in critical illness (223).

Besides peptides hormones like ghrelin, oxytocin and hCG, consideration should be giving to steroid hormones like estradiol and other steroid hormones (1, 6, 224, 225).

## Conclusion

Oxytocin, when discovered 100 years ago, was thought to be produced, stored, and secreted exclusively by the pituitary gland. The target cells for oxytocin were few in number and all were related to pregnancy, parturition and lactation. It is now clear that oxytocin is produced and released at a large number of sites in the body, regulating a broad range of processes, many of which are unrelated to reproduction. Indeed, a major effect of the secreted oxytocin is tempering of pro-inflammatory pathways generated widely by macrophages and other pro-inflammatory cells. Failure to modulate these pathways can lead to severe sepsis including septic shock and death. The control of inflammation by oxytocin is shared with other hormones and hormone-like peptides including vasopressin, ghrelin, hCG and GLP-1. Given the differences in structures, receptors and receptor distribution, we hypothesize that many combinations of anti-inflammatory peptides of native origin will be effective therapies for sepsis. Further clinical studies are required.

## Author Contributions

SM, SP, NM, RK, SS, AA, MF, and JR substantially contributed to the conception and design of the article and interpretation of the relevant literature. DL, CM, HY, KT, and JR added critical intellectual content to the manuscript and can be considered experts on the topic. All authors provided critical feedback and helped shape the research and analysis. All authors contributed to the article and approved the submitted version.

## Funding

The authors declare that this study received philanthropic funding from Alan and Tatyana Forman through Altronix Inc. The funder was not involved in the study design, collection, analysis, interpretation of data, the writing of this article or the decision to submit it for publication.

## Conflict of Interest

Author MJB was employed by Azevan Pharmaceuticals Inc.

The remaining authors declare that the research was conducted in the absence of any commercial or financial relationships that could be construed as a potential conflict of interest.

## Publisher’s Note

All claims expressed in this article are solely those of the authors and do not necessarily represent those of their affiliated organizations, or those of the publisher, the editors and the reviewers. Any product that may be evaluated in this article, or claim that may be made by its manufacturer, is not guaranteed or endorsed by the publisher.
